# Facial Pain Associated with CPAP Use: Intra-Sinusal Third Molar

**DOI:** 10.1155/2014/837252

**Published:** 2014-06-05

**Authors:** Maxime Mermod, Martin Broome, Remy Hoarau, Daniel Zweifel

**Affiliations:** ^1^Service d'ORL, Bâtiment Hospitalier, Rue du Bugnon 46, 1011 Lausanne, Switzerland; ^2^Division de Chirurgie Maxillo-Faciale, Centre Hospitalier Universitaire Vaudois (CHUV), Switzerland

## Abstract

*Objective*. This paper describes a patient with left hemifacial pain elicited by the use of a CPAP mask. *Case Report*. A 74-year-old man was referred with a history of pain in the left maxillary sinus related to the use of his CPAP interface, thereby prohibiting the use of the latter. Computed tomography revealed an intra-sinusal ectopic third molar in the left maxillary sinus floor corresponding to the painful area. After removal of the ectopic tooth under local anesthesia by a Caldwell-Luc approach, the patient was relieved of his symptoms. *Conclusion*. Although an ectopic tooth in the maxillary sinus is rare, this case points out the importance of actively looking for a regional problem if patients cannot tolerate the CPAP interface since this can lead to issues of incompliance and medical complications due to the untreated obstructive sleep apnoea syndrome.

## 1. Introduction

Obstructive sleep apnoea (OSA) is a common medical condition with significant medical consequences. The prevalence of this disease in the United States is currently estimated to be between 5 and 10% [1]. It has been shown that continuous positive airway pressure (CPAP) improves both objective and subjective measures of OSA [2–4]. We describe a case of an otherwise healthy patient presenting with unilateral chronic episodic pain in the region of the left maxillary sinus from an atypical dentoalveolar origin elicited by the use of his CPAP mask, thereby prohibiting its use. To our knowledge, this is the first case of ectopic maxillary third molar presenting as episodic facial pain only related to the air diffusing from the CPAP interface.

## 2. Case Report

A 74-year-old man was referred to the maxillofacial department of our hospital with a long history of chronic episodic facial pain and discomfort arising from the left maxillary sinus. The pain appeared to be related only to the cold air emanating from his full-face mask CPAP interface he used for the treatment of his moderate OSA. His medical history was otherwise unremarkable; in particular there was no history of facial trauma or regional infection and swelling.

Examination revealed mild maxillary sinus pain elicited by percussion in the retromolar area of the left upper quadrant, distal to the only visible molar, which was reactive to cold and not painful to percussion. Nasal endoscopy revealed no purulent discharge.

**Figure 1:**
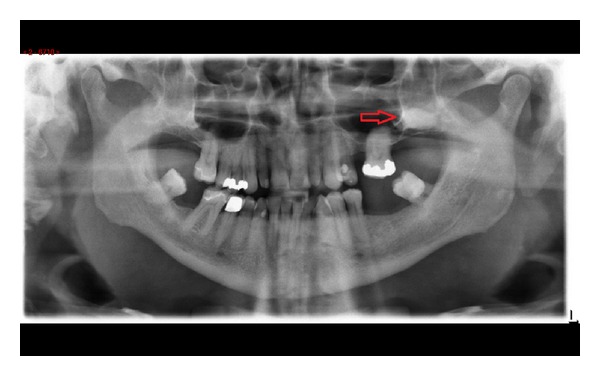
Panoramic radiograph revealing superimposition of left upper third molar and left maxillary sinus (red arrow).

**Figure 2:**
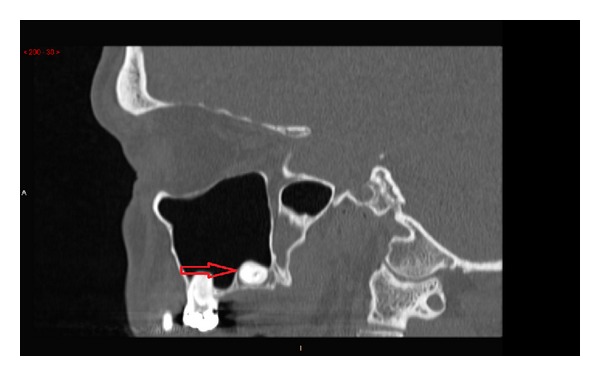
Sagittal view of the CT-scanner showing hyperdense material in the floor of the left maxillary sinus consistent with an ectopic third molar.

An orthopantomogram (OPG) was requested as an overview of the state of the patients' dentition (Figure 1). It revealed radioopacity in the left upper retromolar area superimposed onto the maxillary sinus. A computed tomography (CT) scan was arranged (Figure 2) which showed a hyperdense structure in the floor of the left maxillary sinus which was consistent with an ectopic third molar. It was inverted and presented a complete crown with incomplete root formation. The crown showed either no or very little mucosal covering on its cranial aspect (Figure 2).

**Figure 3:**
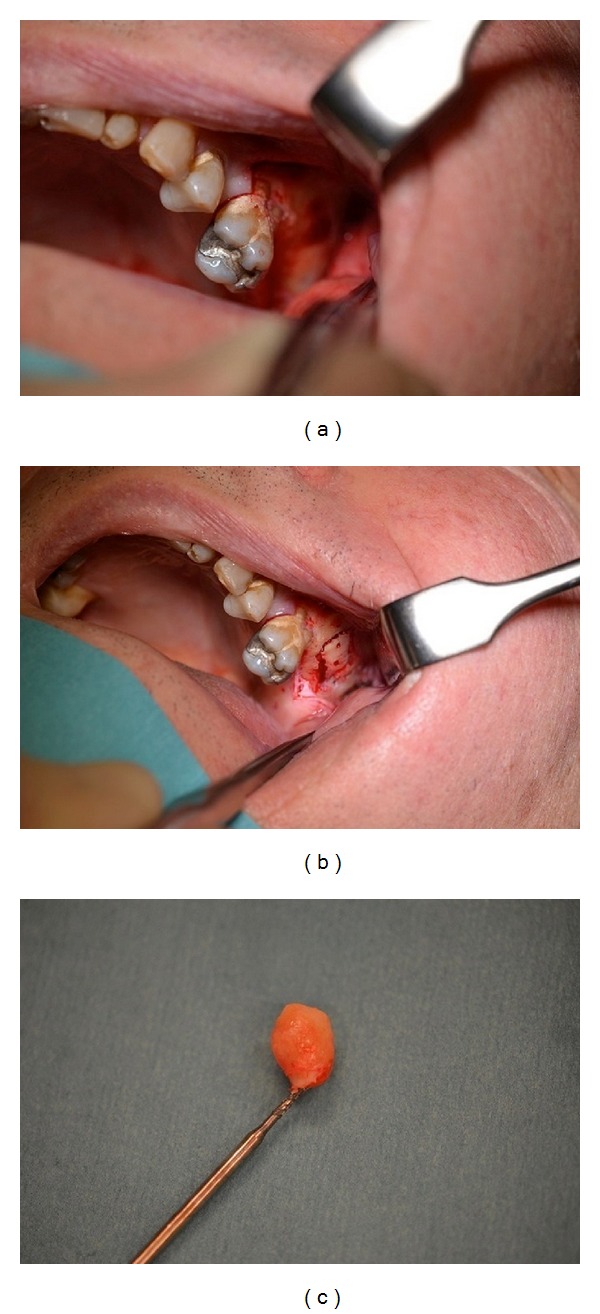
Vestibular incision extending to the left maxillary tuberosity (a), creation of a bony window in the anterior wall of the left maxillary sinus (b), and ectopic third molar showing complete crown but incomplete root formation (c).

After informed consent from the patient had been obtained, we planned an elective procedure under local anesthetic. A mucoperiosteal flap was raised in the region of the permanent first, second, and third molar and extended to the left maxillary tuberosity (Figure 3(a)), a small window to access the sinus was created (Figure 3(b)), and the ectopic tooth was extracted through that window (Figure 3(c)). The surgical wound was closed with 3.0 Vicryl.

Postoperatively, the patient made an uneventful recovery and there was no recurrence of his symptoms in a 3-month follow-up period.

## 3. Discussion

The aetiology of facial pain related to CPAP use can be classified as pressure related or airflow related causes.

Dental or periodontal pain for instance is mainly explained by direct pressure of the device on the gums. This is the case in 15 to 20% of patients treated with CPAP [5]. Abrasion and pain on the ridge of the nose is an issue in 13 to 37% of CPAP users [5].

On the other hand, nasopharyngeal symptoms, like nasal obstruction, rhinorrhoea, sneezing, blocked ears, or excessive mucus, are present in 15 to 65% of the cases. These are more frequent in patients with preexisting problems [6] and appear to be airflow related. The incidence of sinusitis is approximately 8% [6]. More insight in the pathophysiology of those symptoms is needed [7].

The approach to facial pain in CPAP users should not be limited to typical causes of CPAP device related problems. It should encompass classical causes of facial pain such as dentoalveolar pathology, sinusitis, temporomandibular joint disorders, and neuropathic pain [8]. Thus a workup should include a detailed otolaryngological and dental examination.

To date, only 35 cases of ectopic teeth erupting in the maxillary sinus have been reported in the English literature [9]. When erupting into the maxillary sinus, these teeth can present with localised symptoms of sinonasal infection such as nasal discharge, nasal obstruction, facial pain, or fever. Nonetheless, in most instances the condition will remain undiagnosed until it is discovered spuriously due to X-rays performed for unrelated reasons. In our case, the patient demonstrated a cranially displaced and anteriorly rotated tooth with the crown in direct contact with the sinus. Wehypothesize that since the tooth was so exposed, the patient developed dentin hypersensitivity [10] leading to pain and discomfort when the ectopic tooth was subjected to thermal factors such as the cold air of the CPAP.

The eruption of a tooth in nondentate areas is rare, although various cases of teeth erupting in the nasal septum [11], mandibular condyle [12], coronoid process [13], and palate [14] have been reported.

Ectopic tooth formation may happen due to an abnormal interaction between oral epithelium and the underlying mesenchyma during odontogenesis, from pathological processes (cleft palate, infection, bone hyperdensity, and genetic factors) or from trauma [15].

Diagnosis and treatment planning are best made by CT scanner [16]. Surgical access to the maxillary sinus is best achieved by a Caldwell-Luc approach to prevent the complication of oroantral communication [16].

Complete removal of diseased tissue is thought to be mandatory as certain diseases such as cysts or malignancies may coexist with ectopic molars [17]. In asymptomatic cases the patient should be followed periodically with radiographs [18].

The use of CPAP is associated with a reduction in daytime sleepiness and the improvement of quality of life [19]. It is also well documented that successful CPAP therapy results in reduced cardiovascular mortality. Despite the well documented efficacy of the CPAP therapy, adherence to the treatment remains a great challenge [20]. Hence, we should explore every possibility to improve treatment adherence and in cases of associated pain take care to exclude even rare causes.
